# Sildenafil, a Phosphodiesterase Type 5 Inhibitor, Downregulates Osteopontin in Human Peripheral Blood Mononuclear Cells

**DOI:** 10.1007/s00005-017-0455-9

**Published:** 2017-02-16

**Authors:** Beata Kaleta, Agnieszka Boguska

**Affiliations:** Department of Clinical Immunology, Transplantation Institute, Medical University of Warsaw, Poland, Nowogrodzka 59, 02–006 Warsaw, Poland

**Keywords:** Osteopontin, PDE5 inhibitor, Sildenafil, Immune response, Peripheral blood mononuclear cells

## Abstract

The aim of this study was to investigate the ability of sildenafil to regulate osteopontin (OPN) gene and protein in peripheral blood mononuclear cells (PBMCs) from healthy blood donors. OPN is expressed by a wide variety of cell types, including immune cells. OPN functions are linked to various physiological and pathological conditions. Sildenafil is a selective inhibitor of type 5 phosphodiesterase. Sildenafil has recently been found to have immunomodulatory effects in animal models and in studies performed in humans. PMA-stimulated and unstimulated PBMCs from 16 healthy blood donors (men) were cultured with sildenafil (at concentrations of 400 ng/ml and 4 µg/ml). OPN level in culture supernatants was measured by enzyme-linked immunosorbent assay. The analysis of OPN gene expression was performed by real-time PCR. Cell viability was assessed by trypan blue staining. PMA plus ionomycin stimulation of PBMCs resulted in a significant increase of OPN production and gene expression (*p* < 0.001). Sildenafil significantly decreased OPN secretion (*p* < 0.05) and gene expression (*p* < 0.05) in stimulated PBMCs; however, had no effect on OPN in unstimulated PBMCs. Sildenafil did not affect PBMCs viability. Sildenafil downregulates OPN in PBMCs from healthy men. Despite accumulating evidence for the immunomodulatory effects of sildenafil on human immune system cells, further studies are needed to determine if this drug affects the level of cGMP and NF-κB in PBMCs. In addition, it is needed to evaluate sildenafil’s activity in PBMCs from patients with elevated OPN levels.

## Introduction

Osteopontin (OPN) is a member of the small integrin-binding ligand N-linked glycoprotein family proteins (Fisher et al. 2001; Ramaiah and Rittling 2008; Rangaswami et al. 2006). OPN is expressed by a wide variety of cell types such as bone cells, neurons, epithelial cells, pericytes, fibroblasts, hepatocytes, tubular cells, vascular smooth muscle cells and immune system cells [T and B cells, natural killer (NK) cells, NK T cells, dendritic cells, macrophages, neutrophils] (Denhardt and Noda 1998; Ramaiah and Rittling 2008; Uede 2011). OPN is a pleiotropic protein which is involved in physiological tissue remodeling processes [angiogenesis, bone formation and resorption, wound healing (Sodek et al. 2000)]. Moreover, OPN has been implicated in the development of a number of pathological conditions, such as cancer (Afify et al. 2009; Ramaiah and Rittling 2008), autoimmune disorders, infections (Glas et al. 2011; Mishima et al. 2007), asthma (Cantor 1995; Carecchio and Comi 2011; Frenzel and Weiss 2011; Harris and Sadiq 2009; Konno et al. 2011; Murugaiyan et al. 2008; Zandman-Goddard and; Shoenfeld 2003), cardiovascular diseases (Singh et al. 2007), as well as kidney and liver diseases (Cao et al. 2012; Ramaiah and Rittling 2008).

OPN regulates cellular immunity, including innate and adaptive components. OPN stimulates antibodies production by B cells, regulates macrophages migration, activation, capacity for phagocytosis and nitric oxide production and enhances interleukin (IL)-17-producing T helper (Th) 17 cell responses. In addition, OPN induces dendritic cells maturation, promotes activation of T cells, and can enhance the Th1-mediated inflammatory process (Ashkar et al. 2000; Brown 2012; Denhardt and Guo 1993; Murugaiyan et al. 2008; Shinohara et al. 2006; Wang and Denhardt 2008). Moreover, OPN regulates immune suppression, cell adhesion and chemotaxis (Wai and Kuo 2004). These functions are important for the pathological function of OPN.

It is quite a recent finding that some inflammatory processes can be counteracted by phosphodiesterase type 5 (PDE5) inhibitors (Pifarré et al. 2014). Sildenafil is a selective inhibitor of PDE5. PDE5 is a critical component in the cGMP-PKG signaling pathway. cGMP plays an important role in the regulation of activity of a number of cell populations, including inflammatory and immune cells (Chrysant and Chrysant 2012; Szczypka et al. 2012).

At the present time, sildenafil and other inhibitors of PDE5 have Federal Drug Administration approval for the treatment of erectile dysfunction (ED) and pulmonary artery hypertension (Boswell-Smith et al. 2006) but researchers are still looking for new therapeutic indications for these drugs.

Some studies performed in animals and very few observational studies in humans suggest that sildenafil modulates immune system function. Both PDE5 and OPN are expressed in immune cells. Nevertheless, sildenafil’s effects on healthy humans lymphocytes were not assessed. Therefore, the aim of this study was to investigate the ability of sildenafil to regulate OPN expression in peripheral blood mononuclear cells (PBMCs) from healthy men.

## Materials and Methods

### PBMCs Isolation

Ten milliliters of venous blood was collected from 16 healthy blood donors (men). Informed consent was obtained from all individual participants included in the study. PBMCs isolation was performed within 2 h of withdrawal of blood. Blood samples were taken into preservative-free heparin (20 units/ml) tubes, and PBMCs were isolated by centrifugation on Histopaque-1077 (Sigma Aldrich, Germany) of the blood diluted 1:1 with 0.9% sodium chloride (0.9% NaCl, Fresenius Kabi, Germany). PBMCs pellet was resuspended in Parker medium (Biomed, Poland) supplemented with 2 mM l-glutamine (Sigma Aldrich, Germany), 0.1 mg/ml gentamycin (KRKA, Slovenia), β-mercaptoethanol (Sigma Aldrich, Germany), 0.23% Hepes (Sigma Aldrich, Germany) and 10% fetal bovine serum (FBS; Gibco, USA).

### Sildenafil Solution Preparation

Sildenafil citrate salt (Sigma Aldrich, Germany) was dissolved in 0.9% sodium chloride (0.9% NaCl, Fresenius Kabi, Germany) to initial concentrations of 100 and 10 µg/ml.

### Co-culture of PBMCs with Sildenafil and Stimulation with Phorbol Myristate Acetate Plus Ionomycin

PBMCs were seeded at a density of 1 × 10^6^ cells/well in 24-well plates (Greiner CELLSTAR^®^). Sildenafil solutions were added to PBMCs cultures to final concentrations of 400 ng/ml (0.6 µM) or 4 µg/ml (6 µM). Four hundred ng/ml is a serum level of sildenafil after single oral administration (Nichols et al. 2002). Four µg/ml is tenfold higher. Drug concentrations have been selected on the basis of the near therapy doses, according to their pharmacokinetics (Cmax and area under the time-concentration curve). Control cultures contained an equivalent volume of 0.9% NaCl. PBMCs were cultured for 20 h at 37 °C in a sterile environment with 5% CO_2_ and humidified atmosphere. After the incubation period, phorbol myristate acetate (PMA) plus ionomycin (Sigma Aldrich, Germany; 50 ng/ml and 1 µg/ml, respectively) was added to well with PBMCs and two with PBMCs treated with sildenafil. One well with PBMCs and two with PBMCs and sildenafil were not stimulated. Next, PBMCs were incubated at 37 °C in a sterile environment with 5% CO_2_ and humidified atmosphere for 4 h. After the incubation period, culture supernatants were collected and stored at −80 °C for measurement of OPN concentration by enzyme-linked immunosorbent assay (ELISA). PBMCs pellets were lysed with 350 µl of RA1 lysis buffer (NucleoSpin RNA, Macherey&Nagel, Germany) containing 3.5 µl of β-mercaptoethanol (Sigma Aldrich, Germany) and stored at −80 °C for RNA isolation.

### Measurement of OPN Concentration by ELISA

To detect OPN secretion in PBMCs supernatants in response to sildenafil, we measured the concentration of this protein by ELISA according to the manufacturer’s instructions (Human OPN Elisa Kit, Sunred Biological Technology, China) in duplicates. Chromate 4300 Microplate Reader was used for reading at 450 nm. The results were expressed in ng/ml.

### RNA Extraction, Reverse Transcription and Real-Time PCR

Total RNA was extracted from PBMCs with the NucleoSpin RNA kit (Macherey&Nagel, Germany) in accordance with the instructions of the supplier. Total RNA was eluted in a 50-µl volume of RNase-free water. RNA concentration was analyzed by NanoDrop spectrophotometer (ND-1000 Spectrophotometer, NanoDrop Technologies, Inc, USA). 0.1 μg of total RNA was reverse transcribed into cDNA using a commercially available High Capacity cDNA Reverse Transcription Kit (Applied Biosystems, USA) according to the manufacturer’s instruction. Detection of mRNA level in the samples was performed using real-time PCR (RT-PCR) on ABI Prism 7500 Sequence Detector (Applied Biosystems, USA). The analysis of OPN gene expression was performed using a human commercial available assay Hs00959010_m1 (Applied Biosystems, USA). Fluorescence intensities were analyzed using the manufacturer’s software (7500 Software v2.05) and relative amounts were obtained using the 2−∆∆Ct method and normalized for the glyceraldehyde-3-phosphate dehydrogenase (GAPDH).

### Cell Viability Assay

PBMCs were collected by centrifugation and stained with 0.4% trypan blue. Number of total and dead cells was counted using a hemocytometer. Values were expressed as a percentage of control culture (100%).

### Statistical Analysis

All analyses were performed with Statistica version 12.5. Results were analyzed using Student’s *t* test. Data from ELISA and RT-PCR were presented as means ± standard deviations (SD). A probability value of *p* < 0.05 with a 95% confidence interval was considered to indicate a statistically significant difference. All ELISA and RT-PCR analyses were performed in duplicates.

## Results

To investigate the ability of sildenafil to regulate OPN expression in human PBMCs, non-stimulated or PMA + ionomycin-stimulated cells were cultured in the absence or presence of sildenafil at concentrations of 400 ng/ml and 4 µg/ml.

### Cell Viability

Cell viability was assessed by trypan blue staining. Sildenafil had no effect on the viability of PBMCs (percentage of viable cells 98 ± 1.3%; range: 96–99%) in both concentrations.

### Supernatant OPN Concentrations

**Fig. 1:**
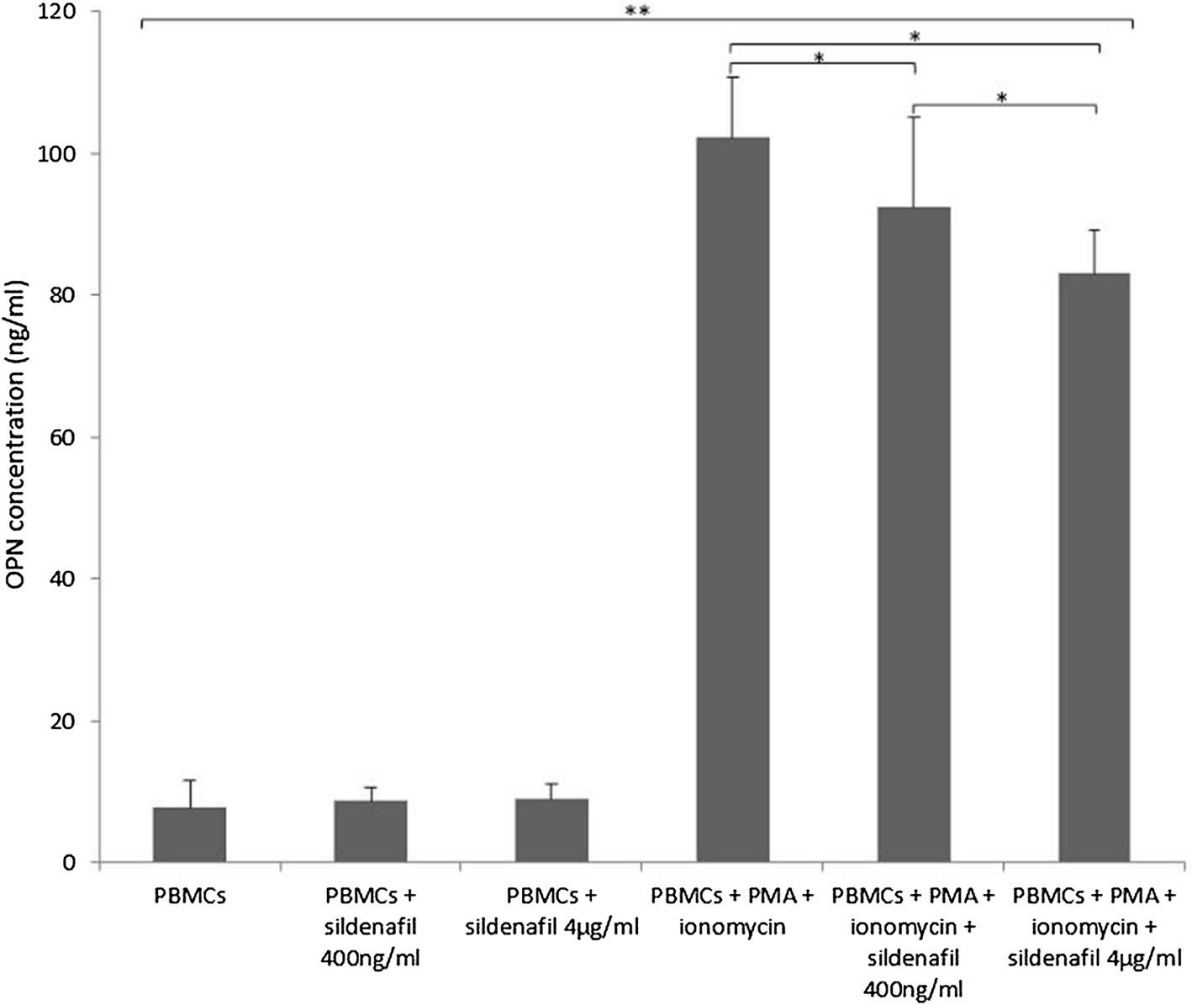
Effects of sildenafil on osteopontin (OPN) production in peripheral blood mononuclear cells (PBMCs). Experiments were performed on phorbol myristate acetate (PMA) plus ionomycin-stimulated and unstimulated PBMCs after incubation with sildenafil (at concentrations of 400 ng/ml and 4 µg/ml). The level of OPN in supernatants was measured by enzyme-linked immunosorbent assay (ELISA, mean ± SD). Statistically significant differences were considered when *p*<0.05 for Student’s *t* test on 16 PBMCs samples. **p < 0.001 (unstimulated PBMCs vs. PMA + ionomycin-stimulated PBMCs with and without sildenafil incubation). *p < 0.05 (PBMCs + PMA + ionomycin vs. PBMCs + PMA + ionomycin + sildenafil 400 ng/ml, PBMCs + PMA + ionomycin vs. PBMCs + PMA + ionomycin + sildenafil 4 µg/ml and PBMCs + PMA + ionomycin + sildenafil 400 ng/ml vs. PBMCs + PMA + ionomycin + sildenafil 4 µg/ml)

Concentrations of OPN in PBMCs supernatants among the different experimental conditions are shown in Fig. 1.

The analysis of OPN concentration in PBMCs supernatants revealed that PMA stimulation resulted in a significant increase of OPN production (*p* < 0.001). We found that sildenafil at both concentrations (4 µg/ml and 400 ng/ml) significantly decreased PMA-induced OPN secretion (*p* < 0.05). Moreover, sildenafil at a concentration of 4 µg/ml significantly decreased the OPN secretion more than at a concentration of 400 ng/ml (*p* < 0.05). However, sildenafil had no significant effects on OPN protein secretion by PBMCs which were not stimulated with PMA plus ionomycin (Fig. 1).

### mRNA OPN Expression by PBMCs

**Fig. 2:**
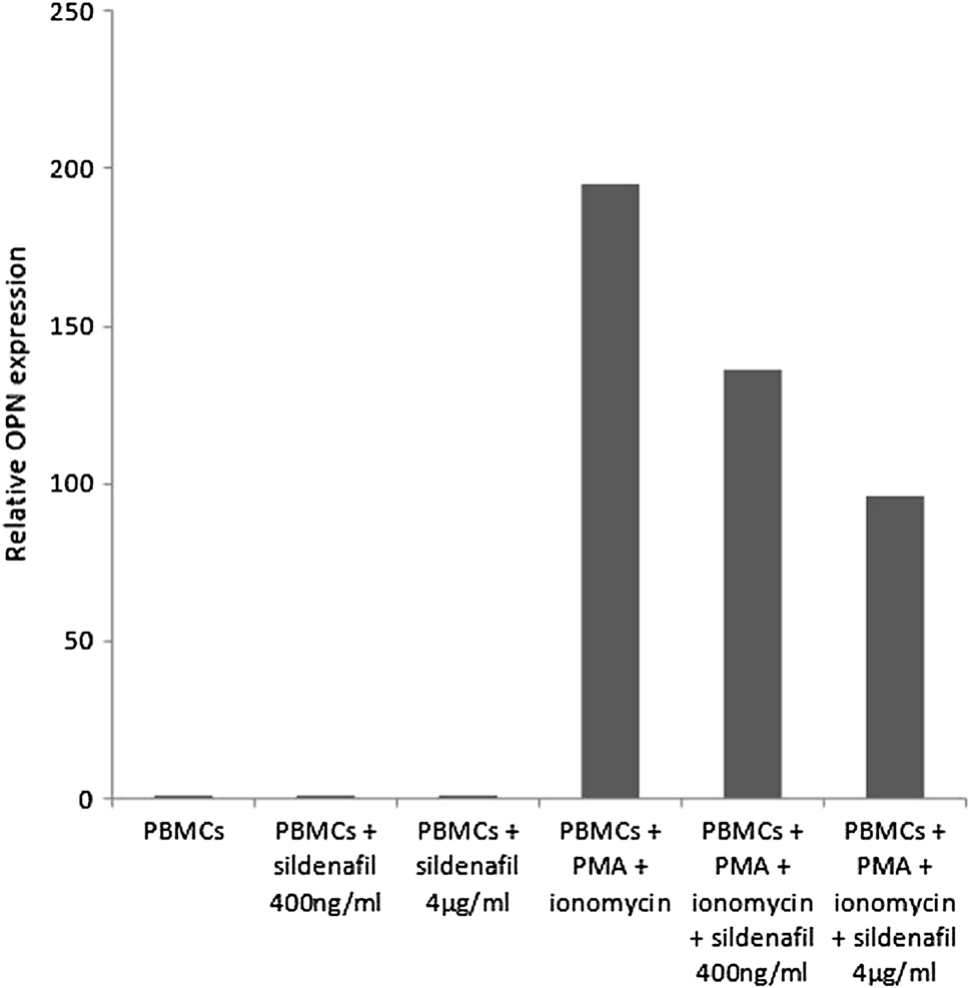
Relative osteopontin (OPN) expression in peripheral blood mononuclear cells (PBMCs) determined by RT-PCR (mean). Experiments were performed on phorbol myristate acetate (PMA) plus ionomycin-stimulated and unstimulated PBMCs after incubation with sildenafil (at a concentrations of 400 ng/ml and 4 µg/ml)

The analysis of OPN gene expression revealed that PBMCs stimulation with PMA plus ionomycin resulted in a significant upregulation of OPN compared to unstimulated PBMCs (*p* < 0.001). Sildenafil at a concentration of 4 µg/ml significantly decreased PMA-induced OPN gene expression (*p* < 0.05). In addition, we observed that sildenafil had no effectt on OPN gene expression in unstimulated PBMCs (Table 1; Fig. 2).

**Table 1:** Effects of sildenafil on osteopontin gene expression in PBMCs

	ΔCt ± SD	*p*
PBMCs	5.403 ± 1.518	
PBMCs + sildenafil 400 ng/ml	5.379 ± 1.008	NS*
PBMCs + sildenafil 4 µg/ml	5.016 ± 1.456	NS*
PBMCs + PMA + ionomycin	–2.207 ± 0.951	
PBMCs + PMA + ionomycin + sildenafil 400 ng/ml	–1.689 ± 0.744	0.06
PBMCs + PMA + ionomycin + sildenafil 4 µg/ml	–1.187 ± 1.267	<0.05**

ΔCt values (mean ± SD) for osteopontin (OPN) in peripheral blood mononuclear cells (PBMCs) determined by RT-PCR after incubation with sildenafil and with and without stimulation with phorbol myristate acetate (PMA) plus ionomycin. Statistically significant differences were considered when *p*˂0.05 for Student’s *t* test on 16 PBMCs samples

*NS* not significant

*PBMCs + sildenafil 400 ng/ml and PBMCs + sildenafil 4 µg/ml vs. PBMCs

**PBMCs + PMA + ionomycin + sildenafil 4 µg/ml vs. PBMCs + PMA + ionomycin

## Discussion

During the last decade it has become clear that inflammation is an important contributor to the development of multiple disorders. Thus, immunomodulatory therapy is beneficial for many diseases.

OPN is expressed by a wide variety of cell types, including immune system cells. OPN is a pleiotropic protein and its functions are linked to various physiological functions and pathological conditions.

Several studies in animal models have shown that sildenafil, a selective PDE5 inhibitor, exerts anti-inflammatory effects but some studies gave inconclusive results.

The study of Pifarre et al. (2011) demonstrated that sildenafil decreased CD3^+^ leukocyte infiltration in the spinal cord in a mouse model of multiple sclerosis and increased forkhead box p3-expressing T regulatory cells (Foxp3 Tregs). However, this drug did not affect production of Th1/Th2 cytokines in mice splenocytes. In another study, Yildirim et al. (2010) evaluated the influence of sildenafil on tumor necrosis factor (TNF)-α and IL-1β production in rats with induced lung fibrosis. The group demonstrated that sildenafil administration decreased serum levels of these cytokines. A strong anti-inflammatory effect of sildenafil was demonstrated in another study conducted in mice (Nunes et al. 2012). It revealed that sildenafil can decrease the levels of TNF-α, interferon (IFN)-γ, IL-2 and IL-1β in animal model of multiple sclerosis. In another study, Pifarré et al. (2014) observed that sildenafil downregulates Th1/Th2/Th17 responses (IL-2, IL-4, IFN-γ) and upregulates Tregs. However, these results were not confirmed by studies of Clayton et al. (2004) and Tsai et al. (2006). In these investigations, sildenafil did not inhibit TNF-α, IL-4 and IL-5 in mice. Karakhanova et al. (2013) evaluated the influence of sildenafil on T lymphocytes in mice. The authors observed that sildenafil treatment has a tendency to decrease the percentage of CD4^+^ and to increase CD8^+^ T cells in male mice. Moreover, sildenafil decreased the serum level of IL-6 in healthy mice (Karakhanova et al. 2013). In addition, it has been observed that application of sildenafil led to a prolonged survival of pancreatic ductal adenocarcinoma (PDAC)-bearing mice, which was due to the decrease in myeloid-derived suppressor cells frequencies and in the systemic vascular endothelial growth factor level. This led to a recovery of T lymphocyte functions and an increase in the frequency of CD4^+^ T cells in tumors and IFN-γ level in serum of PDAC-bearing mice (Karakhanova et al. 2015). In another study conducted in animal model, Schäfer et al. (2009) invastigated the effects of sildenafil on right ventricular remodeling in rats subjected to monocrotaline-induced pulmonary hypertension. The authors observed that sildenafil reduces pulmonary pressure and downregulates markers of hypertrophy and remodeling in the right ventricle, including OPN. A promising role for this PDE5 inhibitor in the modulation of inflammatory processes has also been reported in renal damage in a study of Jeong et al. (2009). The authors observed that in streptozotocin-induced diabetic rats sildenafil treatment may attenuate renal damage by ameliorating oxidative and inflammatory injuries. Sildenafil-treated rats had significantly lower monocyte chemotactic protein-1 (MCP-1) RNA expression. In another study in diabetic mice, Venneri et al. (2015) demonstrated that circulating renal and cardiac Tie2-expressing monocytes (TEMs) are defective in chronic hyperglycemia and that sildenafil normalized their levels by facilitating the shift from classic (M1-like) to alternative (M2-like)/TEM macrophage polarization. These authors suggested that restoration of tissue TEMs with sildenafil could represent an additional pharmacological tool to prevent end-organ diabetic complications.

Moreover, several studies have shown that sildenafil exerts immunomodulatory effects in humans. The aim of a study conducted by Pifarré et al. (2014) was to evaluate sildenafil’s effect on healthy human T effector cells (Teffs). The authors observed that sildenafil significantly decreased IL-2 in Teffs supernatants but changes in IL-4 and IL-13 were not observed. In another study, Di Luigi et al. (2016) evaluated the effects of sildenafil on level of Th1 chemokine CXCL10 in human cardiomyocytes. The group observed that sildenafil significantly decreased CXCL10 in human cardiomyocytes and decreased circulating CXCL10 in patients with diabetic cardiomyopathy. In light of those evidences the authors speculated that sildenafil could be a new tool to control CXCL10-associated inflammation in diabetic cardiomyopathy. The aim of another study conducted by Vlachopoulos et al. (2015) was to investigate the effect of sildenafil on circulating pro-inflammatory markers in ED patients. The authors reported that administration of sildenafil reduced production of fibrinogen, high-sensitivity C-reactive protein, high-sensitivity IL-6 and TNF-α. This study demonstrated for the first time the effect of sildenafil administration on pro-inflammatory markers in men with ED. Several studies conducted in patients with type 2 diabetes demonstrated that chronic treatment with sildenafil is associated with cardioprotection and reduced levels of circulating inflammatory cytokines. Gianetta et al. (2012, 2014) observed that sildenafil reduced concentration of MCP-1 and transforming growth factor-β in patients with type 2 diabetes. Similar study conducted in diabetic patients by Aversa et al. (2008) demonstrated that sildenafil administration reduced markers of vascular inflammation, including the level of IL-6.

The present study evaluated the effects of sildenafil on OPN production in PBMCs from healthy men. Our study demonstrated that sildenafil downregulates OPN gene expression and OPN protein production in PMA-stimulated PBMCs within no effect on unstimulated cells. To the best of our knowledge, this is the first report describing such effects of this PDE5 inhibitor in healthy humans.

PMA activates Ca^2+^/phospholipid-dependent enzyme protein kinases C (PKCs). Activated PKCs regulate multiple physiological processes and activate the NF-κB transcription factors (New and Wong 2007). We could speculate that in PBMCs, high levels of cGMP induced by sildenafil reduce the concentration of Ca^2+^ ions in cytosol, leading to a reduction of PKC and consequently NF-κB activity which contributes to a decreased OPN production. Significantly reduced concentration of OPN in supernatants from unstimulated PBMCs was not observed probably because the sensitivity of the OPN ELISA kit was insufficient to discern the difference when the level is close to the lower end of detection limit.

OPN is a pleiotropic protein which is involved in multiple physiological and pathological conditions. Sildenafil (Viagra^®^ Pfizer) is a relatively commonly prescribed drug, especially for elderly patients whose immune system may weaken as a natural result of aging. It may be the cause of immunologic complications. It is very important to know whether sildenafil can induce immunological changes in healthy organisms. It may reduce the risk of immune system side effects and possible drug-to-drug interactions.

In conclusion, in healthy men PBMCs, sildenafil downregulates OPN production and gene expression. Despite accumulating evidence for the immunomodulatory effects of sildenafil on human immune system cells, further studies are needed to determine if this drug affects the level of cGMP and NF-ĸB in PBMCs. In addition, it is needed to evaluate sildenafil’s effect in PBMCs from patients with elevated OPN levels (type 2 diabetes, cardiovascular diseases, liver diseases, some autoimmune diseases, etc.).
